# The “Multiple Wedge Principle” Applied to the Continuous Method of Dilatation of Stricture of the Urethra

**Published:** 1876-01

**Authors:** John S. Coleman

**Affiliations:** Augusta, Ga.


THE "MULTIPLE WEDGE PRINCIPLE," APPLIED TO THE "CONTINUOUS METHOD" OF DILATATION OF STRICTURE OF THE URETHRA.—*By John S. Coleman, M. D., Augusta, Ga.*—On the 7th of September, 1875, Mr. W., of S. Ca., applied to me for treatment for a stricture of fifteeen years' duration. He has frequently suffered from retention, and oftentimes has had to resort to pressure, *i. e.*, to lie prone across a log, to expel the contents of his bladder. Upon examination I find a stricture in the bulbous portion of the urethra, about half an inch in length, dense, and resisting in its nature. I introduced one of Gemrig's small elastic bougies, "made from the intestines of the silk-worm," and tied it in position.

8th.—Some slight febrile disturbance, which readily yielded to a saline purgative and the usual quantity of quinine, viz., 20 grains daily.

11th.—On removing the bougie, which had now been tied in for eight days, I was surprised to find that the stricture had not yielded in the slightest degree, and that I was unable to introduce the smallest gum-elastic catheter used by Sir Henry Thompson.

12th.—I to-day was again enabled to introduce a No. 2 of Gemrig's scale.

15th.—I now determined to apply the "Multiple Wedge Principle" to the "continuous method" of dilatation, and with that view passed No. 1 down by the side of No. 2, and tied it there.

18th.—I introduced to-day No. 4 of Gemrig's scale by the side of the two already in position.

22d.—Two of Gemrig's No. 1 were now passed along the grooves formed by the instrument in the canal.

As I now felt confident that I could use a good sized catheter, I removed the five bougies, three of which were thickly encrusted with calcareous matter, and introduced a No. 5 bulbous pointed.

25th.—I removed the No. 5 catheter, and tied in a number 10 conical English scale ; this was allowed to remain until the 27th, when I readily introduced a No. 23 steel bougie of Gemrig's scale, the latter corresponding to No. 13 English scale.

I am daily more and more convinced of the superiority of this over all other methods of treating stricture of the urethra, and hope that my professional brethren will give it a trial.

My continued experience in the treatment of stricture corroborates the following language used by Sir Henry Thompson in his work on the urinary organs, p. 26, 11th and 12th lines from the top. The italics are mine : "*First and foremost, dilatation—dilatation always—dilatation without exception, whenever it will succeed.*"—*Medical Record.*

